# Detailed-based dictionary learning for low-light image enhancement using camera response model for industrial applications

**DOI:** 10.1038/s41598-024-64421-w

**Published:** 2024-07-25

**Authors:** Bhawna Goyal, Ayush Dogra, Ammar Jalamneh, Dawa Chyophel Lepcha, Ahmed Alkhayyat, Rajesh Singh, Manob Jyoti Saikia

**Affiliations:** 1 Department of UCRD and ECE, Chandigarh University, Mohali, Punjab 140413 India; 2 Chitkara University Institute of Engineering and Technology, Chitkara University, Rajpura, Punjab India; 3 College of Arts & Science Applied Science University, Manama, Kingdom of Bahrain; 4 College of Technical Engineering, The Islamic University, Najaf, Iraq; 5 Department of ECE, Uttaranchal Institute of Technology, Uttaranchal University, Dehradun, 248007 India; 6 Department of Electrical Engineering, University of North Florida, Jacksonville, FL 32224 USA

**Keywords:** Dictionary learning, Camera response function (CRF), Image enhancement, Research, Technology, Innovative, Engineering, Electrical and electronic engineering

## Abstract

Images captured in low-light environments are severely degraded due to insufficient light, which causes the performance decline of both commercial and consumer devices. One of the major challenges lies in how to balance the image enhancement properties of light intensity, detail presentation, and colour integrity in low-light enhancement tasks. This study presents a novel image enhancement framework using a detailed-based dictionary learning and camera response model (CRM). It combine*s* dictionary learning with edge-aware filter-based detail enhancement. It assumes each small detail patch could be sparsely characterised in the over-complete detail dictionary that was learned from many training detail patches using iterative ${{\ell}}_{1}$-norm minimization. Dictionary learning will effectively address several enhancement concerns in the progression of detail enhancement if we remove the visibility limit of training detail patches in the enhanced detail patches. We apply illumination estimation schemes to the selected CRM and the subsequent exposure ratio maps, which recover a novel enhanced detail layer and generate a high-quality output with detailed visibility when there is a training set of higher-quality images. We estimate the exposure ratio of each pixel using illumination estimation techniques. The selected camera response model adjusts each pixel to the desired exposure based on the computed exposure ratio map. Extensive experimental analysis shows an advantage of the proposed method that it can obtain enhanced results with acceptable distortions. The proposed research article can be generalised to address numerous other similar problems, such as image enhancement for remote sensing or underwater applications, medical imaging, and foggy or dusty conditions.

## Introduction

In recent years, image processing systems have been broadly utilised in a variety of industrial activities, including video monitoring, industrial manufacturing, remote sensing monitoring, and intelligent transportation, due to the rapid advancement of computer vision technologies, and plays a significant role in daily life, industrial production, military applications, etc.^1^. Nevertheless, during the image acquisition process, there are often some uncontrollable elements present in the environments which leads to several image degradation problems. Notably, in poor-light environment such as indoors, nighttime, or foggy days, the lighting reflected from the object surface could be feeble, and thus photograph quality may be severely degraded by noise and colour distortions^2^. Processing, transmission, storage, and other operations further deteriorate the already degraded images. As the name suggests, low-light describes ecological settings with below-average illumination. All images captured in weak lighting conditions fall under the general term of low-light images. However, it is thus far impossible to find the explicit theoretical parameters that characterise low light settings in real-world appliances, and so no unified standard exists. Hence, every manufacturer of image sensors has their own standards. The images captured in different environments characteristically have low contrast, poor illumination, colour distortion, a higher noise ratio, etc. The use of software algorithms that provide a flexible option and enhance low light image quality and videos through image processing has long been a foremost field of study. Thus, it is significant and practical benefit to study low-illumination image enhancing techniques for refining the functionality of imaging systems. The main aims of enhancing poor light photographs include improving global and local image contrast, visual perception, and suitability for human and computer processing by preventing noise amplification and ensuring adequate real time performance. In order to produce clear photographs or videos, it is crucial to increase the dependability and utility of data collected in low-light conditions. The expansion of image information mining depends on improving the consistency and quality of outdoor photographic systems by improving the reliability of images with subjective pictorial perceptions of individuals and streamlining computer vision equipment’s analysis and processing of such images. In addition to studies on subjects like marine image investigation and foggy image clarity, the findings of related investigations can be broadly utilised in domains like traffic monitoring, outdoor video capture, military aviation research, etc. Low-light image enhancement^2^, a significant area of study in the domain of image processing has interdisciplinary appeal, innovative, a broad scale of potential applications, and has recently attracted interdisciplinary research attention. For quite some time, researchers from all over the world have been paying significant attention to this domain^3,4^.

Zhuang et al.^5^ presented an innovative method where the histogram function is generated to make the histogram curve smooth to avert information loss in the processed images. Adaptive Gamma adjustment for histograms is introduced to enhance illumination by using the histogram function. Also, the log-exp transformation method is proposed, which steadily improves low-magnitude while maintaining high magnitude while falling. Hao et al.^6^ developed a Retinex-based enhancement technique with effective semi-decoupled Retinex image decomposition. Based on the Gaussian total variation algorithm, an illumination layer *I* is precisely computed using the source image $S$ only, while the reflectance *R* is simultaneously computed using intermediate* I*. Moreover, image noise can be reduced while $R$ is being estimated. We recommend the reflection technique and PCA as tools for image enhancement. The multiscale concept and reflection model form the foundation of this strategy which adapts to dark images^7^. Before being converted to HSV colour space, a source colour image is stretched for removing any colour chromatic distortions. The illumination component of the V channel is determined utilising the notion of multiscale principle. Further, an image brightness enhancement technique based on Fechner theory is used to robustly adjust the parameters of enhancement function. A technique for illumination and reflectance decomposition based on the pixel level nonlocal Haar transform was developed by Hou et al.^8^. An illumination coefficient is reconstructed using a unique low frequency component from the Haar transform for every analogous pixel set and the reflectance coefficient is restored using the remaining high frequency components. An image is slightly sharpened during the image brightness enhancement process due to the perfect resemblance of pixels like the pixel groups and the discrete Haar transform, which enable highly adequate image disintegration. A novel self-calibrated illumination (SCI) learning system was designed by Ma et al.^9^ for rapid, adaptable, and reliable image enhancement in real low-light environments. To do this, the technique constructs a weight-sharing cascaded illumination learning system. This strategy develops a self-calibrated scheme that allows convergence across outputs of each phase, leading to benefits that only require a single basic block for interpretation while considerably decreasing computational cost. Also, an innovative strategy explored one-to-many interactions using a normalising flow model^10^. An invertible network learned to transform the distribution of generally exposed photographs into a Gaussian distribution using low-light shots as the condition. As a result, it is possible to accurately describe the conditional distribution of the normally exposed photographs, and the training procedure for the invertible network’s enhancement direction is like being limited by the loss function that more accurately captures complex structural details of photographs. A reliable technique for enhancing medical images was established by Lepcha et al.^11^ based on the morphological processing of residuals using a special kernel. This system integrates nonlinear technology as a linear low pass filter to identify important regions where edges will be precisely preserved. The original shape of the edges can be restored by fusing the restored regions with a result of low-pass filtering. This method provides an observer with a clearer and less noisy image that can be easily interpreted. A competent semantically contrastive learning paradigm was recently proposed^12^. Beyond an existing low light enhancement (LLE), it forms an image enhancing operation through multitask joint learning. Another enhancing method for low-light images was introduced by Sun et al.^13^. This technique initially produces dual replicas of the original image. After that, structural details are extracted from the texture using the relative total variation scheme to obtain the irradiation element of the original image. This is merged with multistage Retinex for extracting the reflectance element of the original photograph. Then, both aspects of the original shape are concurrently enhanced utilising bilateral gamma function corrections, histogram equalisation and filtering. Singh et al.^14^ established a technique in order for estimating the illumination of LLE. The three main aims of this method are to estimate the structure-aware illumination, enhance estimated illumination and adjust the final brightness of the enhanced illumination.

Deep learning-based image processing models are emerging rapidly nowadays^15–17^. A trainable hybrid network is proposed by Ren et al.^18^ for improving the perceptibility of low illumination photographs. The proposed network consists of two separate streams that work together to simultaneously provide an overall structure as well as information about the enhanced image. In general, a network of decoders and encoders is used by the information stream to compute the global information of the low light input. This method proposes to use Zero-DCE^19^, which formulates image enhancement as a challenge for image explicit curves that are estimated using deep network. To forecast high order and pixel-by-pixel curve for dynamic range correction of the source images, this method constructs a thin deep network. The key focus was placed on the pixel value range, monotonicity and differentiability when producing the curve estimate. In order to improve low illumination images, Yang et al.^20^ introduced a semi supervised learning method. The recovery of linear band representations of boosted images from paired normal or low light images is proposed. The given linear bands are then reconstructed using a different learnable linear transformation based on an adversarial learning scheme driven by perceptual quality using unpaired data to get this enhanced representation. A recent deep learning network (DLN) took advantage of the recent development of CNNs^21^. A number of lightning back projection blocks (LBPs) compose the proposed deep learning network. The LBPs iteratively perform darkening and brightening operations to learn the residual from normal illumination estimations. This method further introduced a feature aggregation block that adaptably aggregates the outputs of many LBPs to fully utilise the local and global properties. Zhu et al.^22^ introduced two parallel branches for enhancing low-light images by learning several forms of correction. In particular, the two branches directly forecast pixel offsets to enhance local details and build global transformation curves to enhance overall contrast. Additionally, the technique generates a differentiable histogram loss, which offers guidance on the overall contrast. Hu et al.^23^ proposed a dual stage unsupervised technique that divides enhancement problem into a pre-enhancing as well as post refinement issue. The low light image is initially pre enhanced utilising a traditional Retinex-based algorithm. To further enhance the image quality, it employs a refinement network trained using adversarial training in the second stage. We introduced a novel context-sensitive decomposition link to address issues with the two-stream method for picture improvement^24^. The spatially varying lightning guidance is introduced to achieve the edge aware smoothness property of the lighting element. A competent end to end attention guided technique based on multi-branch CNN for enhancing images was established by Lv et al.^25^. It first constructs a simulated database using carefully crafted low illumination modelling models. This database is far larger and highly diversified compared to those that are already available. Further, it learns dual attention maps for controlling the tasks of enhancing intensity as well as denoising using a novel dataset as training data. Zhao et al. developed a generative method for Retinex disintegration, where disintegration is considered a generative concern^26^. The Retinex decomposition is performed by the RetinexDIP without the aid of any outdoor images and enhancement is carried out using swiftly adjusted approximated illumination. Further, PRIEN was proposed based on neural networks^27^. The main concept involves employing a recursive unit made up of a residual block and a recursive layer for unfolding the source image to extract features periodically. EnlightenGAN, a more efficient unsupervised GAN was developed by Jiang et al.^28^, where the network is trained with image pairs and shows excellent generalisation on the range of test images captured in the real world. The proposed strategy promises to regularise unpaired training utilising data collected from the input itself rather than supervising the learning with ground truth data. It also recommends numerous enhancements, such as the attention mechanism, self-regularised perceptual loss fusion and global–local discriminator structures for low light enhancement tasks.

A competent unsupervised disintegration and correction network for enhancing low illumination images was introduced recently^29^. This technique draws its inspiration from the Retinex model, which first divides images into their light and reflectance components and does not use paired data for training. An illumination correction network (ICN) processes the decomposed illumination after which it fuses it with reflectance to get an initial boosted output. To split a photograph into reflectance and illumination layers, Wu et al.^30^ present a Retinex-based deep unfolding network that unfolds an optimisation issue for the learnable network. The three learning related schemes that are accountable for data dependent initialisation, extremely effective unfolding optimisation and user defined illumination are meticulously built by formulating the disintegration concern and an implicit priors regularised method. A competent model for enhancing low light images was introduced recently^31^. Using these three subnets, it performs decomposition, denoising, enhancing contrast and maintaining details. This method utilises both frequency information and spatial data of an image to maintain the characteristics while enhancing the contrast. A dual stage unsupervised model to improve low illumination photographs is established by Xiong et al.^32^. The method recommends an illumination aware denoising method at the stage of noise suppression in order to minimise actual noise at various locations under the effect of lighting conditions. The method generates false triplet samples to enable unsupervised training and proposes an adaptive content loss to maintain contextual information. Fan et al.^33^ developed an enhancement method for improving low illumination photographs. Liu et al.^34^ present a LAENet for low illumination image enhancement. Liu et al.^34^ present a LAENet for enhancement of low illumination images. This method openly rethinks the spatial-frequency property of human vision, and actual research is performed on the correlation between spatial frequency, receptive field size, and light enhancing properties. Jiang et al.^35^ introduced DEANet, a special Retinex-based convolutional neural network for improving low-light photographs. Wang et al.^36^ introduced a BrightFormer in enhancing low illumination photographs that incorporates convolutions and transformers. Some of the main findings of this study include the fusion of local and global information using spatial and channel attention in feature equalisation. Further, the use of gated parameters and illumination based prior knowledge in self-attention will improve feature expression flexibility and make it simpler to extract global features. As a result, local features are preserved, eliminating useless features.

In this paper, we present a low light image enhancement scheme based on detailed-based dictionary learning and a camera response model. The dictionary training and sparse reconstruction are the first two steps in the detailed-based dictionary learning process. Then, using a training pairs of detail patches obtained from high-quality images, we train the over-complete detail dictionary by progressively lowering a ${\ell}1$-norm energy function. We use the training dictionary to restore the increased detail layer during the reconstruction stage. We also provide a gradient guided optimisation technique to increase local coherence between patches. In order to get enhancement outcomes that maintain naturalness, we also consider in-camera processing when creating enhancement algorithms. The pixel value and light input do not typically have an inverse relationship in digital cameras. The camera response function (CRF) is a nonlinear function that connects the pixel value of an image with the irradiance of the camera sensor. We present CRF-based paradigm for improving low-light images. There are two main steps to the process; first optimise a camera response model (CRM) and its parameters. According to our study, designing a reliable algorithm to determine the three-color channel response curves utilising just low illumination images is difficult. Thus, we used a suitable fixed response curves as a substitute to retrieve the model parameters. We confirm that these approximations do not introduce any notable quality drops in results for most cameras. Furthermore, we utilise lighting estimation algorithms to compute an exposure map that contains the desired exposure ratio for each pixel.

The main contributions of the proposed method are summarised as follows:

- This study introduces dictionary learning for image enhancement, which employs sparse reconstruction and dictionary training to improve image quality.
- This strategy incorporates the Retinex model, and the camera response model while local exposure adjustments are performed for low-illumination images to obtain high-quality images.
- The proposed method demonstrates how to achieve better results with fewer distortions using reasonable fixed camera response curves (CRCs) rather than computing precise three-channel response curves.
- In addition to image enhancement of low-light images, the proposed algorithm can be utilised to perform other applications such as medical image enhancement, enhancing underwater images, remote sensing image enhancement, and dusty weather image enhancement.

The remainder part of the study is structured as follows. The related works are briefly illustrated in “Related work” section. The proposed image enhancement method using detailed based dictionary learning and a camera response model is summarised in “Methods” section. “Experimental results” section exhibits comprehensive experimental details, results, and discussion using publicly available datasets. “Conclusions” section concludes our work and illustrates some potential future work.

## Related work

This section illustrates an edge-aware filter based on guided image filter, dictionary learning, camera response function (CRF) and brightness transform function (BTF) which is relatable to our enhancement method.

### Edge-aware filter based on a guided image filter

In contrast to conventional filters however, edge aware filters are a set of special image manipulation strategies because of their accuracy to image structures. These filters appropriately maintain image details by decomposing images into their base and detail layers. He et al.^37^ introduced fast and non approximate linear time filter that generates good quality filtering results while considering information from a guiding image also known as a guided image filter. This filter represents a translation variant filter based on a local linear model. It consists of three parts, an input image I, a guidance image G and an output image O. The basic requirements for this filtering are as follows; (a) the total linear result represents the linear transform of the guidance image G, and (b) a guidance image G is as identical as possible to the input image I. The initial conditions show that:

1 $$ {{\text{O}}_{\text{i}}}  = {{\text{a}}_{\text{k}}} {{\text{G}}_{\text{i}}}  + {{\text{b}}_{\text{k}}} \quad \forall {\text{i }} \in {\upomega _{\text{k}}} , $$

where ${\upomega }_{\text{k}}$ represents a square window of dimension $\left(2\text{r}+1\right)\times \left(2\text{r}+1\right)$. The local filter model assures that a resultant image O has edges only in the regions where a guidance image also has one, since $\nabla \text{O}=\text{a}\nabla \text{G}$. In ${\upomega }_{\text{k}}$, the linear components $\left({{\text{a}}_{\text{k}}},{{\text{b}}_{\text{k}}}\right)$ are fixed. They can be calculated by minimising a cost function E, or by minimising the squared difference between the resultant image O and the input image I in the window ${\upomega }_{\text{k}}$ as follows:

2 $${\text{E}}\left( {{\text{a}}_{\text{k}}} ,{{\text{b}}_{\text{k}}} \right) = \sum\limits_{{{\text{i}} \in \omega_{{\text{k}}} }} {\left( {\left( {{\text{a}}_{\text{k}}} {{\text{G}}_{\text{i}}} + {{\text{b}}_{\text{k}}} - {{\text{I}}_{\text{i}}}  \right)^{2} + \varepsilon {{\text{a}}_{\text{k}}}^{2} } \right)} ,$$

where $\varepsilon$ represent regularisation factor panelising larger ${\text{a}}_{\text{k}}$. The components $\left({{\text{a}}_{\text{k}}},{{\text{b}}_{\text{k}}}\right)$ can be computed instantly using linear regression^37^. The value of ${\text{O}}_{\text{i}}$ in Eq. (1) depends on window where pixel $i$ may be present in many (overlapping) windows ${\upomega }_{\text{k}}$. It can be described by averaging over all probable values of ${\text{O}}_{\text{i}}$ as

3 $$ {{\text{O}}_{\text{i}}}  = \overline{{\text{a}}_{\text{i}} } {{\text{G}}_{\text{i}}}  + \overline{{{{\text{b}}_{\text{i}}} }} , $$

where $\overline{{\text{a} }_{\text{i}}}$ and $\overline{{\text{b} }_{\text{i}}}$ represent mean components across all overlapping window $i.$ Despite the linear components $\overline{{\text{a} }_{\text{i}}}$ and $\overline{{\text{b} }_{\text{i}}}$ fluctuate spatially, their gradient can be lesser than G near robust edges. Thus, we have $\nabla \text{O}=\overline{\text{a}}\nabla \text{G }$, which indicates sudden intensity shifts from a guidance image are still mostly maintained in the resultant image O. Equation (3) gives a description of the guided image filtering. When a source image also acts as a guidance image, a guided filter acts similar to bilateral filter. However, this filter prevents gradient reversal artefacts since it is an edge-preserving function with great computing efficiency. The edge aware filters are broadly utilised in image processing as well as computer visions such as detail enhancement, image smoothing, detail extraction, non-photorealistic rendering, and others.

### Dictionary learning

For instance, dictionary learning^38^ has been shown to be a sophisticated learning method in image processing and compute vision, where it has been used abundantly for a variety of applications including image restoration^39^, image super-resolution^40^, image denoising^41^ and others. With the use of a dictionary of the basic components learned from the images, it splits the image into the basic components. The two important stages in the method are the training dictionary and the computation of sparse coefficients to represent signals utilising image dictionary components. In this study, edge-aware filter-based detail enhancement is first integrated with a dictionary learning. It assumed that each smaller detail patch may be sparsely characterized in the over complete detail dictionary from numerous training detail patches. As a result, by mapping the visibility restriction of training detail patches into enhanced detail patches, the dictionary learning may successfully address a variety of appearance concerns in the procedure of enhancing details. Once there is training pairs of better-quality images, we can use trained dictionary to restore the enhanced detail layers and generate an enhanced output with a clear visibility. In the training stage, we first employ some advanced cameras to collect enough high-quality photos. Then we randomly extract a lot of small patches from these images with photographic appearances and simply compute their local intensity differences as detail patches. Finally, we construct a training set of detail patches and train an overcomplete detail dictionary by iteratively minimizing an ℓ1-norm energy function. The impact of images of different camera exposures on dictionary learning is negligible. Since we employed advanced cameras with HDR mode to collect these high-quality photos. Their HDR mode can take multi-exposure photos in a scene to compose a high dynamic range^42^.

### Camera response function (CRF) and brightness transform function (BTF)

Numerous computer vision algorithms assume that the scene irradiance is accurately recorded by the image intensity. To enhance the visual quality of acquired photographs, camera manufacturers nevertheless consistently employ various non-linear camera techniques, like while balance as well as demosaicing. Thus, ignoring these processes may result in a reduction in algorithmic efficiency for algorithms that need to determine scene attributes like irradiance and illumination^43^. The camera response function (CRF) has been introduced to design those non-linear camera procedures. As a result, CRF determines the relationship between pixel values (P) and image irradiance (E).

4 $${\text{P}} = f\left( {\text{E}} \right),$$

where $f$ represents a nonlinear CRF function. An irradiance E to the camera sensor can change linearly as the settings of camera exposure get adjusted. But the intensity of image P may not alter linearly in numerous situations due to the nonlinear in-camera systems.

As a result, a mapping function between several exposure photographs could also be non-linear function. The mapping function is known as BTF. It explains the correlation between two images ${\text{P}}_{0}$ and ${\text{P}}_{1}$ that were captured at different exposures while in the same scene as

5 $${\text{P}}_{1} = g\left( {{\text{P}}_{0} ,k} \right),$$

where *k* represents an exposure ratio and $g$ is a BTF. The characteristics of camera imaging processing are defined by CRF and BTF. The camera response model is made up of these two functions. By means of using the descriptions of CRF and BTF, we get the equation shown below as

6 $$g\left( {f\left( {\text{E}} \right),k} \right) = f\left( {k{\text{E}}} \right).$$

Above equation is known as comparametric. It explains a bond between $f$ and $g$. The two functions could be mutually converted using the comparametric equation. In Ref.^44^, it makes three assumptions to determine the similarities between $f$ and $g$. First, $f$ is constant for all pixels on the sensor. Next, a range of $f\left(\cdot \right)$ could be normalised to $\left[\text{0,1}\right]$. Lastly, $f$ increases monotonically. Under these assumptions, define $\mathcal{F}:\left|\text{0,1}\right|\to \left|0,1\right|$ as a hypothetical space of $f$ as

7 $${\mathcal{F}}: = { }\left\{ {f\left| {f\left( 0 \right) = 0, f\left( 1 \right) = 1, x } \right\rangle y \Leftrightarrow f\left( x \right) > f\left( y \right)} \right\}.$$

We observed that BTF and CRF share the same common features according to Eq. (6).

## Methods

In general, most of the state-of-the-art algorithms extract image detail and then boost it to produce an enhanced appearance. The suitability of obtaining and enhancing the detail layer such as construction of advanced camera determines the appearance effect in the images^42^. Here the typical enhancement model could be determined using following function as:

8 $$E = I + d^{*} = I + n \times d,$$

where $I$ represent an input image, $\text{d}^{*}$ represent an enhanced detail layer, d represent a main detail layer and $n$ indicate a scaler factor where it is set to 5 in our study and E represent an initial enhanced result. In Eq. (8), the detail layer is produced by edge aware filter (as discussed in “Edge-aware filter based on a guided image filter” section).

### Detailed-based dictionary learning

We often restore visual quality using the detail dictionary trained using a large number of higher quality images. We considered an enhanced detail patch could sparsely be characterized in the adequately selected over over-complete dictionary. Based on hypothesis, we further recommend the new learning-based enhancement approach described below:

9 $$\text{d}_{\text{x}}^{*}  = \text{nd}_{\text{x}}  \approx \sim \text{Da}_{\text{x}}, $$

where x represents a small patch of an image while ${{d}_{x}^{*}}\epsilon {\mathbb{R}}^{N}$, and N represent an amount of pixels in each small patch x, ${\text{D }} \in { }{\mathbb{R}}^{{{\text{N}} \times {\text{K}}}}$ represent an over complete dictionary trained from detail patches sampled from higher quality photographs, where K represent number of atoms in dictionary D, α represent coefficient vector of sparse representation for some α ∈ ${\mathbb{R}}^{\text{k}}$ with ${{\Vert \alpha \Vert }_{0}}\ll \text{K}.$ Equation (9) represent the enhanced detail patch ${{\text{d}}_{\text{x}}^{*}}$ which specifies a sparse linear combination relating to detail dictionary with K atoms and α_x_ indicates vector with very less $\left( { \ll {\text{N}}} \right)$ non zero entries. Undoubtedly, a novel model focusses on finding the solution for sparse components. It is an NP hard optimisation concern since the optimised components must be adequately sparse. We define a ${\ell_{1}}$-norm minimization function using a trained dictionary to balance the sparsity solution and limit the approximation to the enhanced detail layer ${{\text{nd}}_{\text{x}}}$:

10 $$ {\upalpha _{\text{x}}^{*}}  = \mathop {\arg \min }\limits_{\upalpha _{\text{x}}} \left\| {{\text{D}}{\upalpha _{\text{x}}}  - {{\text{nd}}_{\text{x}}}} \right\|_{2}^{2}  + \uplambda \left\| {\upalpha _{\text{x}}} \right\|_{1} , $$

where ${{\upalpha}_{\text{x}}^{*}}$ represent ideal sparse components and $\lambda$ denote balance parameter between fidelity and sparsity. As soon as the components have been optimally executed, a novel enhanced detail layers could be effectively restored as $d_{x}^{*} = D{{\upalpha }_{\text{x}}^{*}}$. In Eq. (10), an optimisation and reconstruction should carry an over-complete dictionary that has been pretrained from significant number of sampled detail patches.

In dictionary learning, adequate high-quality enhanced photographs are required according to Eq. (9). The low-quality images are often manually improved in conventional method and the yields their enhanced results. The boosted results are simulated and insufficient to create a perfect training set because manual enhancement is difficult to maintain a consistent quality. Thankfully, certain advanced cameras offer HDR modes that enable many images to be taken simultaneously at several exposure levels and combined into a higher quality HDR image. We will be able to gather a lot of training photographs that are of photographic as a result rather than then enhancement results of manual modification. Further, the training pairs can be successfully formed by sampling from these superior quality images. First, we arbitrarily extract the large number of small patches from the training pairs ${\text{P}} = \left\{ {{\text{L}}^{1}} ,{{\text{L}}^{2}} , \ldots ,{{\text{L}}^{\text{m}}}  \right\}$ to simulate the photographic enhancing results and then we simply calculate local intensity difference in order to create a training pairs of detail patches ${\text{Y}} = \left\{ {{\text{y}}_{1}}, {{\text{ y}}_{2}{ , \ldots ,{{\text{y}}_{\text{n}}} |{{\text{y}}_{\text{x}}} = {{\text{L}}_{\text{x}}^{\text{t}}}} - {\text{mean}}\left( {{\text{L}}_{\text{x}}^{\text{t}}} \right),{\text{ t }} \in { }\left[ {1,{\text{m}}} \right]} \right\}$. Where the dictionary learning should completely assure component sparsity obtained by ℓ1-norm minimisation in Eq. (10). We determine a new ℓ1-norm minimisation function for solving detail dictionary and ensure component sparsity as

11 $$D = \mathop {\arg \min }\limits_{D,\alpha } \left\| {Y - D\alpha } \right\|_{2}^{2} + \lambda \left\| \alpha \right\|_{1} ,$$

where each column of D can eliminate scaling uncertainty using $\ell_{2}$-norm constraint $\left\| {{\text{D}}_{\text{i}}} \right\|_{2}^{2} \ll 1,{\text{ i }} \in \left[ {1,{\text{K}}} \right];{\text{the }}\ell_{1}$-norm constraint of α is to ensure sparsity, λ represent a balance parameter between dual items of function set to 0.1. In Eq. (11), an optimisation generally performed is an alternate manner over two unknowns D and $\alpha$. We first utilise a Gaussian random matrix to initialise a detail dictionary D with normalised columns. Next, we correct the dictionary D and modify sparse component α using a minimisation practice as shown below:

12 $$\alpha = \mathop {\arg \min }\limits_{\alpha } \left\| {Y - D\alpha } \right\|_{2}^{2} + \lambda \left\| \alpha \right\|_{1} ,$$

where linear programming could be used to resolve this issue successfully. Finally, we repair the sparse coefficients α and revise the detail dictionary D using $\ell_{2}$-norm minimisation function.

13 $${\text{D}} = \mathop {{\text{argmin}}}\limits_{{\text{D}}} \left\| {{\text{Y}} - {\text{D}}\upalpha} \right\|_{2}^{2} .$$

This problem is considered as classic example of quadratically restricted quadratic programming. Then, until step Eq. (11) of the optimisation problem converges, we iterate between Eqs. (12) and (13). We have so far successfully completed training procedure and retrieved an adequate detail dictionary D. The dictionary will be used to enhance low-light photographs with a high-quality appearance.

### Sparse reconstruction

It intends to boost the overall visual quality of enhanced output of dictionary learning. In Eq. (10), it specifies that early enhanced detail layer is significant for reconstruction in addition to trained dictionary. Thus, the detail layer is extracted using a rapid and non-approximate linear time filtering^37^, then directly multiply a scale factor $n$ in order to boosting it. Furthermore, other edge aware filters can be utilised for generating a detail layer if we need to maximise their enhancing impacts. Next, all the preparations for sparse reconstruction are complete that includes detail dictionary and early enhanced detail layer.

**Figure 1:**
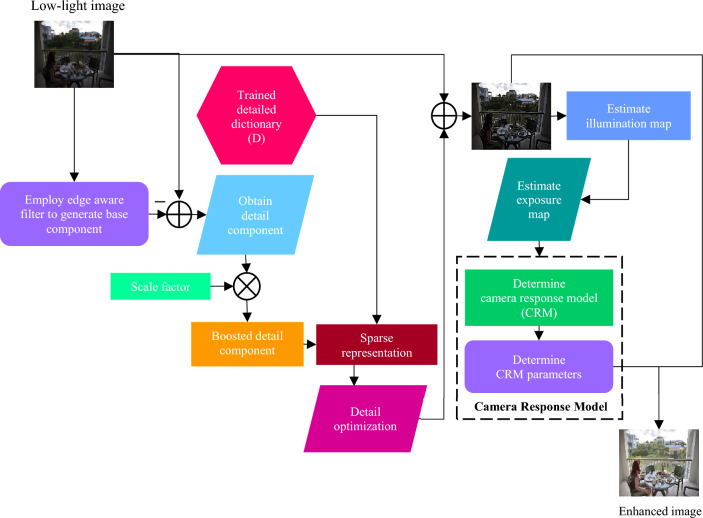
Flowchart of the proposed image enhancement method.

Theoretically, using a detail dictionary D as well as early enhanced detail ${{\text{nd}}_{\text{x}}}$ in the small patch x, we first optimise Eq. (10) to generate its sparse coefficients ${{\upalpha }_{\text{x}}^{*}}$. Second, we assemble each restored detail patches ${{\text{d}}_{\text{x}}^{*}} = {\text{D}}{\upalpha_{\text{x}}^{*}}$ and merge it to the entire detail layer ${\text{d}}^{*}$. Lastly, we design a gradient guided optimisation operator to continuously enhance local enhancement cohence.

14 $$\widehat{{d^{*} }} = \mathop {\arg \min }\limits_{{\widehat{{d^{*} }}}} \left\| {\widehat{{d^{*} }} - d^{*} } \right\|_{2}^{2} + \lambda_{1} \nabla \left\| {\widehat{{d^{*} }} - \nabla g} \right\|_{2}^{2} ,$$

where $\widehat{{{\text{d}}^{*} }}$ represent optimised detail layer and $\nabla {\text{g}}$ represent a guided gradient that is generally correlated to input image and ${\uplambda }_{1}$ represent regularisation parameter, we set 0.05 in our study. However, we merely achieve a perfect guided gradient since local coherence is not certain following conventional detail enhancement. When compared to ${\text{nd}}$, we can see that an input image contains optimal local coherence. So, we select its gradient as average guided gradient and set a small value ${\uplambda }_{1}$ in order to balance local coherence and enhancing impact. The optimised detail layer $\widehat{{{\text{d}}^{*} }}$ is combined to obtain the enhanced result $E = I + \widehat{{{\text{d}}^{*} }}.$ The schematic flow diagram is shown in Fig. 1. An optimised detail by Eq. (14) can considerably boost local coherence and generate a high-quality image.

### Camera response model (CRM)

In this study, we compute an enhanced image, where images collected from the similar camera in the similar environments with constant intensity conditions. The traditional Retinex algorithm adopts that the amount of light reaching the observers could be divided into two portions.

15 $${\text{E}} = {\text{R }} \circ {\text{ T, }}$$

where $\left( {{\text{R}},{\text{T}}} \right)$ represents scene reflectance map and the illumination map. ${\text{E}}$ represents an amount of light that reaches the camera sensor i.e., image irradiance. While $\circ { }$ stands for element-wise multiplication function. As per Eq. (4), the CRM and camera irradiance model E could be utilised to obtain input low illumination image P.

16 $$P = f\left( E \right) = f\left( {R \circ T} \right).$$

As earlier mentioned, an enhanced image ${\text{P}}^{\prime}$ is expressed by

17 $$P^{\prime} = f\left( { R \circ 1} \right).$$

In above equation, 1 indicates a matrix where all components are ones. We can describe a relationship between P and ${\text{P}}^{\prime}$ byor

$$P^{\prime } = f\left( R \right)\overset {Eq.15} \leftrightarrows f\left( {E^\circ \left( {1{ \oslash }T} \right)} \right),$$

18 $$P^{\prime } = g\left( {f\left( E \right),1{ \oslash }T} \right)\overset {Eq.6} \leftrightarrows \Leftrightarrow g\left( {P,1{ \oslash }T} \right),$$

where ${ \oslash }{ }$ indicates elementwise division. The output ${\text{P}}^{\prime}$ are produced by adjusting an exposure of source image P according to the Eq. (18). Thus, the output image could be expressed as

19 $$P^{\prime} = g\left( {P, 1{ \oslash } T } \right) = g\left( {P,K} \right).$$

However, for instance we replace P by L as an input image which is an initial enhanced output from the dictionary learning and sparse reconstruction (“Detailed-based dictionary learning” and “Sparse reconstruction” sections). Therefore Eq. (19) is further re-written as

20 $$P^{\prime} = g \left( {P, 1{ \oslash } T } \right) = g\left( {L,K} \right).$$

In contrast to constant exposure ratio $k$ in Eq. (5), an exposure ratio K in Eq. (20) represent a matrix that specifies the desirable exposure ratio for each pixel. The exposure ratio map K is defined as

21 $$K = 1{ \oslash }{\text{ T}}{.}$$

Further, this process mainly splits into two stages based on Eq. (20): (a) First, determine a suitable CRM and its parameters (b) Next, estimate an exposure matrix K for ensuring all pixels reach the desirable exposures.

#### CRM determination

We make no assumptions about the employment of camera response models in our work. Yet, the effectiveness of different camera response models differs. Some models with suitable functional forms can accurately describe the response properties of most real-world cameras, while others cannot. Therefore, we must use a suitable camera response model for our study to enhance the efficiency of the proposed strategy. Here, we present a few popular models before evaluating how well they function. The camera response curves (CRCs) could be utilised simply for improving low illumination photographs without the assumption of any functional forms of BTF and CRF. The computer stores CRCs as sampling points. In accordance with Eq. (6), we could determine the scatter of BTF for a specific exposure ratio ${\text{k}}$ utilizing the subsequent formula as

22 $$g\left( {L,k} \right) = f\left( {k f^{ - 1} \left( L \right)} \right).$$

Thus, an enhanced image ${\text{P}}^{\prime}$ can be computed utilising interpolation strategies like spline interpolation. Besides utilising camera response curves directly, numerous computer vision algorithms including radiometric calibration can benefit from assuming the functional forms of BTF or CRF. Some articles make camera response models assumption using functional version of CRF $f$. A two-parameter Sigmoid model on the basis of human vision system that assumes CRF in Ref.^45^:

23 $$f\left( E \right) = \left( {1 + \alpha } \right)\frac{{E^{b} }}{{E^{b} + \alpha }},$$

where *a* and $b$ are model parameters. As per the Eq. (6), the BTF for the Sigmoid could be determined by

24 $$g\left( {L,k} \right) = \frac{{k^{b} L\left( {1 + \alpha } \right)}}{{\left( {k^{b} + 1} \right) + 1 + \alpha }}.$$

In contrast to Sigmoid which has a static number of parameters, numerous techniques proposed models with a diverse number of parameters to fulfil many application requirements. Those models utilise numerous bases. A linear combination of the basis is then used to express the CRF as

25 $$f\left( E \right) = f_{0} \left( E \right) + \mathop \sum \limits_{n = 1}^{N} {c_{n}} {h_{n}} \left( E \right),$$

where N indicates number of parameters, ${{\text{c}}_{1}} , \ldots ,{{\text{c}}_{\text{M}}}$ is a model parameter. The computing inverse CRF ${\text{f}}^{ - 1}$ closed-form solutions for these models are often difficult. Hence, it is equally challenging to retrieve the functional forms of BTF. Therefore, utilising these models, we required to sample CRF and obtains CRCs at initial to enhance low-light photographs. The enhanced images and BTF curves are then computed.

The trigonometric model^44^ and polynomial model^46^ could be observed as utilising the similar base function ${{\text{f}}_{0}} \left( {\text{E}} \right): = {\text{E }}$ with variable basis h_n_(E). A polynomial model utilises basis ${{\text{h}}_{\text{n}}} \left( {\text{E}} \right): = {{\text{E}}^{{\text{n}} + 1}} - {\text{E}}$, whereas the trigonometric model make use of half sine basis ${{\text{h}}_{\text{n}}} \left( {\text{E}} \right): = {\text{sin}}\left( {{\mathrm{n\pi \,E}}} \right)$. By investigating CRCs^44^, proposed a EMoR empirical model. The real-world response curves from DoRF dataset^44^ were used to derive the eigenvectors of the curves using principal component analysis (PCA). A CRF of particular camera could be then denoted using Eq. (25) with ${\text{f}}_{0} \left( {\text{E}} \right): = {\text{f}}_{0}$ and ${{\text{h}}_{\text{n}}} \left( {\text{E}} \right): = {{\text{h}}_{\text{n}}}$ while ${\text{f}}_{0}$ indicate average curve of DoRF dataset and ${{\text{h}}_{\text{n}}}$ indicate *n*-th eigen vector. By making an assumption of the functional form of BTF $g$ enables to obtain camera response models. Contrary to when computing BTF $g$ utilising an assumption of CRF $f$, there are uncertainties while utilising an assumption of $g$ for estimating $f$^47^. In order to eliminate these uncertainties, one must either assume on the exposure ratio $k$ or on the form of CRF $f$. Gamma correction is a method that is often used to make images lighter or darker. This approach allows one to express BTF on real-world cameras as an exponential function.

26 $$g\left( {L,k} \right) = P^{\gamma } ,$$

where γ represent model parameter based on exposure $k$. The CRF of Gamma correction in Ref.^48^ is assumed as

27 $$f\left( E \right) = e^{{\left( {E^{\alpha } } \right)}} ,$$

when parameter a satisfies $k^{a} = \gamma$. As per Eq. (27), a CRF of gamma correction do not pass the origin which means it does not satisfy the hypothetical space $f$ in Eq. (7). Consequently, employing gamma correction to enhance photographs might not be acceptable^48^. A few image enhancing algorithms, like LIME^49^ and DEMEF^50^, makes the assumption that BTF is a linear function. For instance, LIME recommends that enhanced image ${\text{P}}^{\prime}$ could be computed by

28 $$ {\text{P}}^{\prime }  = {\text{ }}\left( {\frac{1}{{\text{T}}}} \right)^{{\text{c}}} {\text{L}}. $$

As per Eq. (20), the BTF of LIME can be expressed by

29 $$g\left( {L,k} \right) = \beta L,$$

where ${\upbeta } = {{\text{k}}^{\text{c}}}$ = *kc.* Thus, utilising Eq. (6) and the hypothetical space of $f$, we can obtain its CRF by

30 $$f\left( E \right) = E^{c} ,$$

while the CRF fulfils the basic requirements of CRF hypothetical space, these models assume a linear BTF which may not be the case for several cameras. As a result, distortions may be present in the resultant enhanced photos. The beta gamma correction suggested in Ref.^51^ could be observed as a development of gamma correction and LIME. In order to improve low illumination photographs, a two-parameter CRM is proposed. A BTF of beta gamma correction can be expressed as

31 $$g\left( {L,k} \right) = \beta L^{\Upsilon } ,$$

where $\left( {\beta ,\gamma } \right)$ factors are based on exposure K. After assumptions and inferences, we retrieve a CRF of beta gamma as

32 $$f\left( E \right) = \left\{ {\begin{array}{*{20}l} {e^{{b\left( {1 - E^{\alpha } } \right)}} } \hfill & { if \;\Upsilon \ne 1} \hfill \\ {E^{c} } \hfill & { if\;\Upsilon = 1} \hfill \\ \end{array} } \right.,$$

where ${\text{a}} = {{\text{log}}_{\text{k}}} \Upsilon ,$ ${\text{b}} = \frac{{{\mathrm{ln\beta }}}}{1 - \Upsilon }$ and ${\text{c}} = {{\text{log}}_{\text{k}}} {\upbeta }$***.*** When γ = 1, the BTF and CRF of beta gamma correction is similar to LIME. Further in beta gamma correction, when γ ${ } \ne$ 1, the BTF and CRF are two parameter nonlinear functions. In Ref.^43^ presents the most popular camera response models and the capability of these models are studied. An important indicator for CRMs is the competence of fitting the real-world camera CRCs. The real-world CRCs from DoRF dataset are fitted using CRF in each camera model. By resolving the following optimisation concern, an optimal parameter of each CRT model is determined by

33 $$\arg \min \mathop \sum \limits_{i = 1}^{M} \left( {L_{i} - f\left( {E_{i} } \right)} \right)^{2} ,$$

where $\left( {E_{i} ,{{\text{L}}_{\text{i}}} } \right)$ represents *i*-th sampling points of specific CRCs and M represent a total number of sampling points. This study utilises a sampling practice from Ref.^52^ in order to resolve the optimisation concern in Eq. (33). A goodness of fit for every model is computed by RMSE. In general, LIME, Sigmoid, and EMoR get reasonably decent outputs, which specifies that they are more accurate than other models at obtaining enhanced results. The three-parameter EMoR model and the two-parameter Sigmoid model almost has similar perfoance. The exposures of underexposed photographs are then adjusted using these models. To determine camera response curves through multiple exposure photographs, the method by Ref.^53^ is used. The four representative models are selected in order to use Eq. (33) for fitting the obtained curves. The well exposed images are estimated employing a specified exposure ratio for enhancing the low light photographs. In general, the output of response models such as beta gamma correction and LIME exhibits apparent distortions. This is due to these two models fails to adequately determine the response properties of camera. Less distortions may be seen in case of Sigmoid and EMoR models.

#### Determination of model parameters

The parameters of the camera response model must be established for each image. As earlier stated, CRF determine the response properties of cameras. Thus, variety of cameras hold different CRCs. Depending on the cameras, the response curves for three colour channels are also diverse. The three-channel response curves $\left\{ {{\text{f}}_{\text{r}}} ,{{\text{f}}_{\text{g}}} ,{{\text{f}}_{\text{b}}} \right\}$ for each camera should theoretically be determined. However, it is challenging to precisely define the three-channel curves $\left\{ {{\text{f}}_{\text{r}}} ,{{\text{f}}_{\text{g}}} ,{{\text{f}}_{\text{b}}} \right\}$ for this study. Often, we only have low illumination source image and don’t know the camera settings. There are numerous techniques introduced to get camera response curves^54^, but these techniques might not be very trustworthy if the quality of source image is relatively poor. For the theoretical example, we use two sample from single image CRF algorithm^55^ for estimation of $\left\{ {{\text{f}}_{\text{r}}} ,{{\text{f}}_{\text{g}}} ,{{\text{f}}_{\text{b}}} \right\}$ from low light photographs and simultaneously improve the poor-quality image utilising the estimate curves. These studies employ traditional techniques and CNN networks. It is clear that there are obvious distortions in the results obtained utilising the curves computed by these techniques. Also, the three-channel channel curves $\left\{ {{\text{f}}_{\text{r}}} ,{{\text{f}}_{\text{g}}} ,{{\text{f}}_{\text{b}}} \right\}$ need to be estimate for each input image since we do not know the camera settings which reduces efficiency. As a result, we approximate real curves by using a suboptimal CRCs of the real-world response curves in DoRF dataset ${\tilde{\text{f}}}$ instead computing of $\left\{ {{\text{f}}_{\text{r}}} ,{{\text{f}}_{\text{g}}} ,{{\text{f}}_{\text{b}}} \right\}$. Then, the model parameters can be determined by resolving the optimisation problem in Eq. (33).

#### Estimation of exposure ratio map

**Figure 2:**
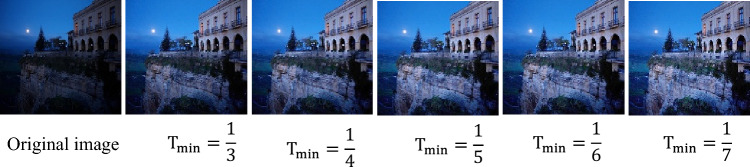
Input image and enhanced results using Eq. (34) with different ${\text{T}}_{\text{min}}.$

An exposure ratio map ${\text{K}}$ is estimated in this section. As already specified earlier, K and illumination map T are inversely proportional. Hence, we first determine T and then compute K. The computation of scene illumination from the photographs is an ill-posed concern. A presumption that illumination T of the scene is an edge preserving as well as texture removing low frequency range is one that is often employed to tackle this problem. The illumination of for image processing task can therefore be estimated using a variety of method^56^. To assist to find T in this paper, we use speed-up solver presented in Ref.^49^. The illumination map T is first obtained and further refined exposure ratio map K is tentatively determined utilising Eq. (21). Nevertheless, a signal-to-noise ratio (SNR) will reduce for most of the photographs when illumination level reduces. The generated photographs will contain a significant amount of noise if a pixel with a very low SNR score is given a larger exposure ratio value. The denoising procedures can be used as a post processing strategy however doing so will make our approach less effective. Hence, we set a maximal exposure value for pixels with extremely low illumination.

34 $$K\left( x \right) = \frac{1}{{\max \left( {T\left( x \right),T_{\min } } \right)}},$$

where ${\text{T}}_{\text{min}}$ represent a threshold value. We consider that pixel with illumination less than ${\text{T}}_{\text{min}}$ has very less SNR score and assigned it a fixed exposure ratio $\frac{1}{\text{T}}_{\text{min}}$. Figure 2 shows an example case. It is observed that the ${\text{T}}_{\text{min}}.$ plays an important role in image enhancement task.

In general, the enhanced image will exhibit visible noise, which degrades the quality of the image if a maximum exposure ratio value is not set. While the maximum exposure ratio may diminish visibility in extremely dark regions, it enhances the photographic perception of the enhanced results by reducing image noise significantly. After computing the exposure ratio map K*,* we apply K in Eq. (20) to obtain the final enhanced results.

## Experimental results

**Figure 3:**
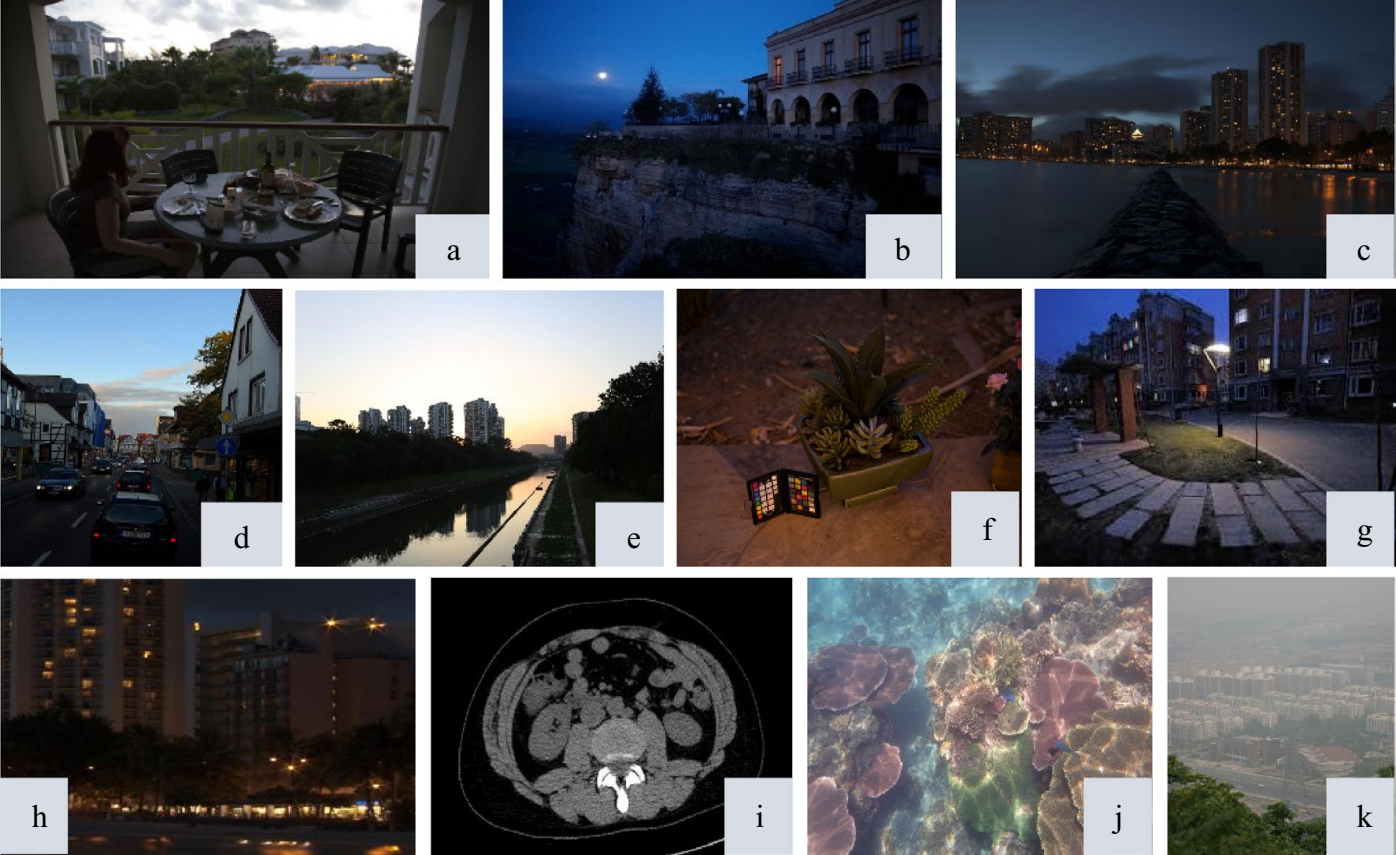
Eleven sample images are used for presentation. These are represented as (**a**–**j)**.

We compare our proposed method with several existing techniques by conducting an extensive experiment with both subjective and objective aspects. Our method is compared with numerous competing techniques, including semi-decoupled decomposition (SDD)^6^, attention guided image enhancement (AGIE)^25^, progressive recursive image enhancement network (PRIEN)^27^, unsupervised image enhancement using decoupled networks (UIE-DN)^32^ and context sensitive decomposition network (CSDNet)^24^. To properly measure our technique, we tested it on images of numerous scenes. We use the publicly available dataset of LIME^49^, BSDS500^57^, NPE^58^, Flickr^59^, UIEB^60^ and 2016 NIH-AAPM-Mayo Clinic Low Dose CT Grand Challenge^61^ for evaluation and validation of the proposed algorithm. The total of 11 images are considered for presentations in this study, as shown in Fig. 3. For a quantitative comparison, we utilised feature similarity index (FSIM)^31^ along with some recently proposed metrics, including measure of enhancement (EME)^42^, mean absolute error (MAE)^31^, entropy^62^, visual information fidelity (VIF)^63^ and average brightness (AB)^64^. All indicators tend to favour higher numbers with the exception of MAE and AB. We also conducted a parameter study to evaluate the effects of scale factor, regularisation parameter and local window radius on the proposed method.

### Experimental details

We train the overcomplete dictionary, which requires roughly 30 min for dictionary training by sampling 100,000 patches and using iterative optimisation. In contrast, sparse reconstruction for all small patches takes between 100 and 300 s at diverse resolutions (400 × 300, 600 × 480, and 800 × 600). In general, a guided filter^37^ is used to generate an initial boosted detail layers which are then added to sparse reconstruction. Our method uses the Sigmoid camera response model. We apply the Sigmoid model to fit a suboptimal curve of the DoRF dataset and recover model parameters *a* = 0.6 and *b* = 0.9. In the case of the exposure map solver *K*, we utilise the default parameters of LIME for solving an illumination map T and set ${\text{T}}_{\text{min}} = \frac{1}{7}$ to retrieve K in all experiments. The parameters regularisation parameter $\left( \lambda \right)$, scale factor $\left( n \right)$ and local window radius $\left( r \right)$ are set to 0.05, 5, and 16, respectively. In addition, the parameter *σ* are fixed to 2. These experimental settings normally produce better results. In all experiments, we utilise default settings presented by their respective authors for all other competing methods. All the simulations are carried out in Matlab R2021b on a PC running with Windows 10 OS with 16 GB of RAM and a 3.5 GHz CPU. The study trains two detail dictionaries, one with 512 atoms and the other with 1024 atoms. We first degrade the detail layers of the high-quality images to create low-quality versions of the 200 source images. We use two separate dictionaries to get enhanced results from these low-quality images. We also create a testing set of 100 images, comprising 90 genuine high-quality images and 11 enhanced by our model. On top of that, we selected the 11 enhanced images from the testing set by organising 15 testers to compare their appearances.

### Results and discussion

**Figure 4:**
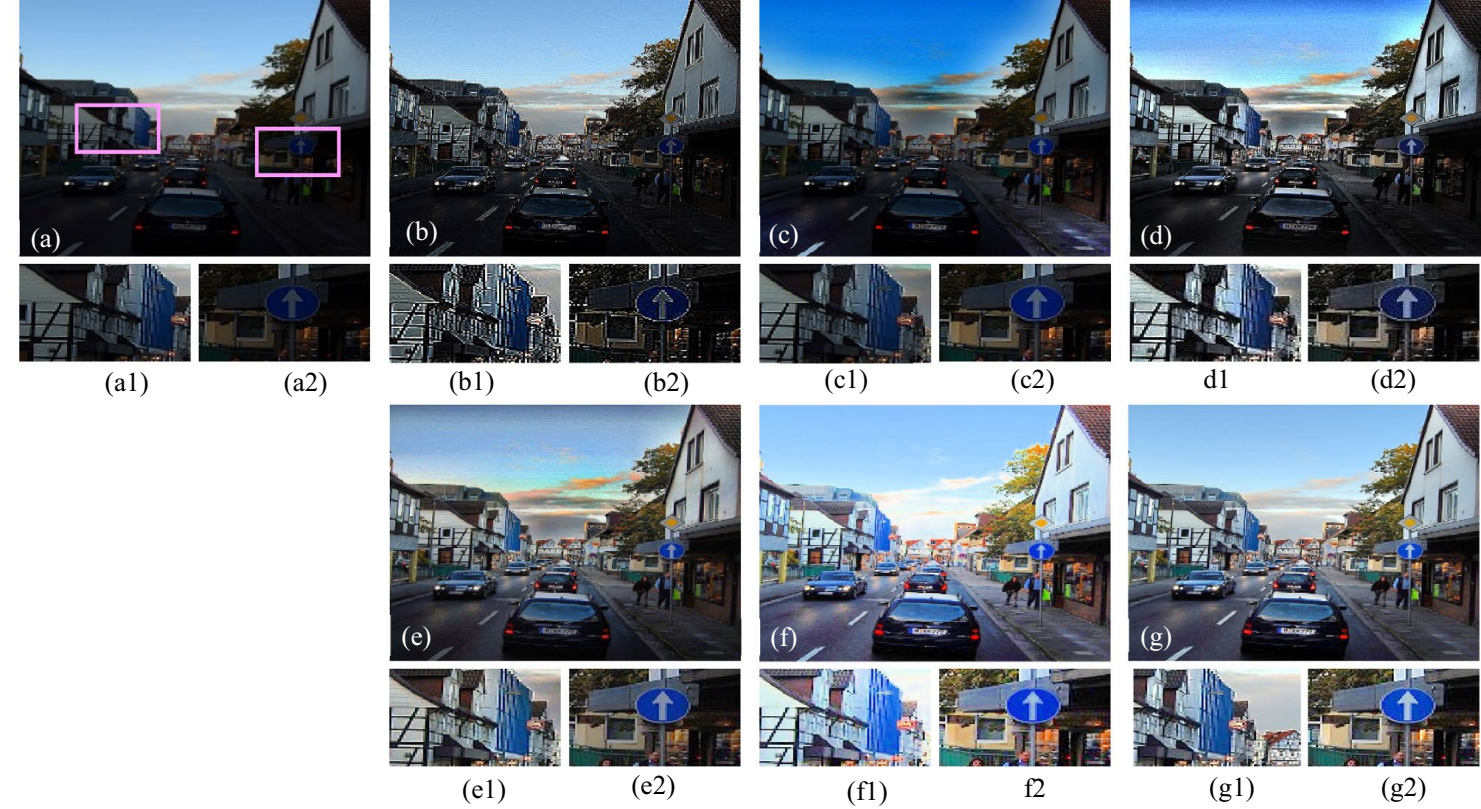
Comparison of enhancement results for test image “*d*”. (**a**) Input, (**b**) SDD^6^, (**c**) AGIE^25^, (**d**) PRIEN^27^, (**e**) UIE-DN^32^, (**f**) CSDNet^24^ and (**g**) Proposed method.

**Figure 5:**
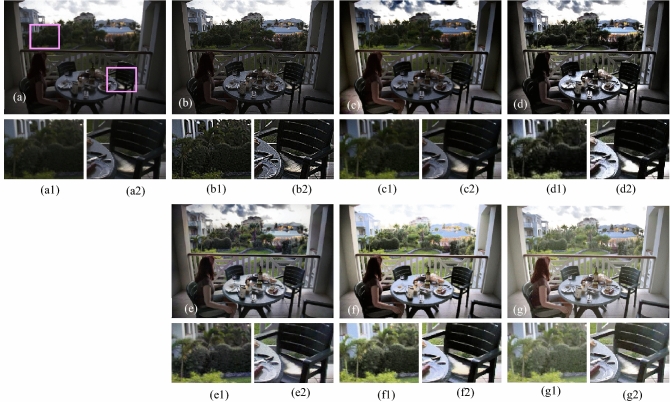
Comparison of enhancement results of test image “*a*” (**a**) Input, (**b**) SDD^6^, (**c**) AGIE^25^, (**d**) PRIEN^27^, (**e**) UIE-DN^32^, (**f**) CSDNet^24^ and (**g**) Proposed method.

**Figure 6:**
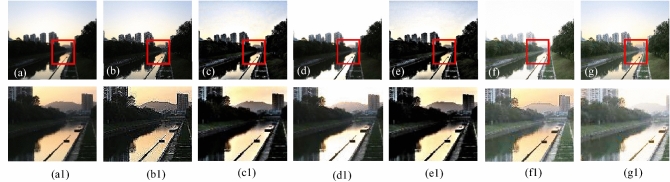
Comparison of enhancement results for test image “*e*” (**a**) Input, (**b**) SDD^6^, (**c**) AGIE^25^, (**d**) PRIEN^27^, (**e**) UIE-DN^32^, (**f**) CSDNet^24^, and (**g**) Proposed method.

**Figure 7:**
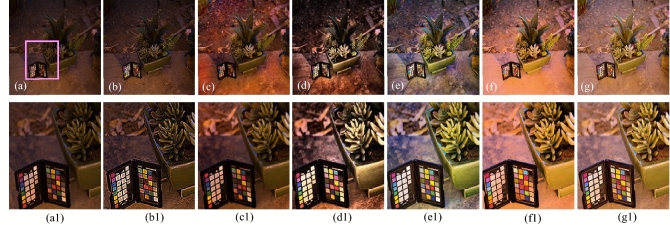
Comparison of enhancement results for test image “*f*”. (**a**) Input, (**b**) SDD^6^, (**c**) AGIE^25^, (**d**) PRIEN^27^, (**e**) UIE-DN^32^, (**f**) CSDNet^24^ and (**g**) Proposed method.

**Figure 8:**
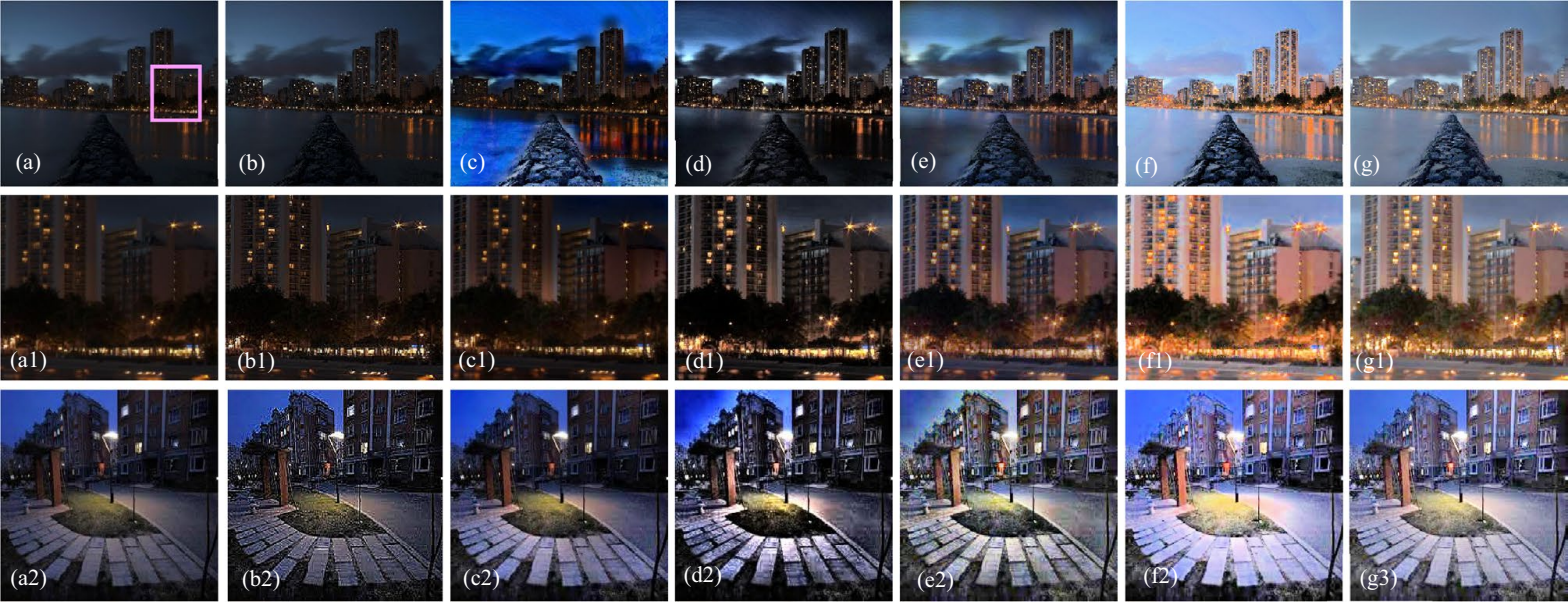
Comparison of enhancement results for test images “*c*”, “*h*” and “*g*”. (**a**) Input, (**b**) SDD^6^, (**c**) AGIE^25^, (**d**) PRIEN^27^, (**e**) UIE-DN^32^, (**f**) CSDNet^24^ and (**g**) Proposed method.

**Figure 9:**
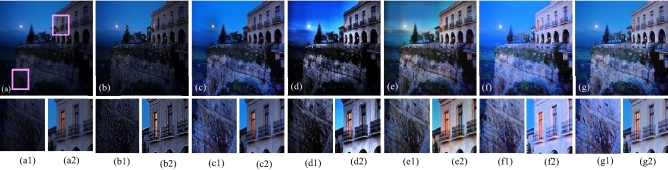
Comparison of enhancement results for test image “*b*”. (**a**) Input, (**b**) SDD^6^, (**c**) AGIE^25^, (**d**) PRIEN^27^, (**e**) UIE-DN^32^, (**f**) CSDNet^24^ and (**g**) Proposed method.

**Figure 10:**
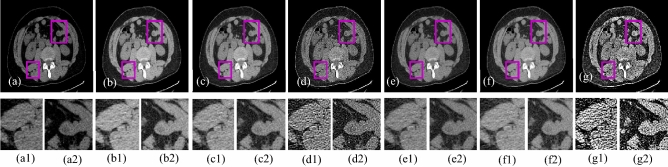
Comparison of enhancement results for medical image “*i*”. (**a**) Input, (**b**) SDD^6^, (**c**) AGIE^25^, (**d**) PRIEN^27^, (**e**) UIE-DN^32^, (**f**) CSDNet^24^ and (**g**) Proposed method.

**Figure 11:**
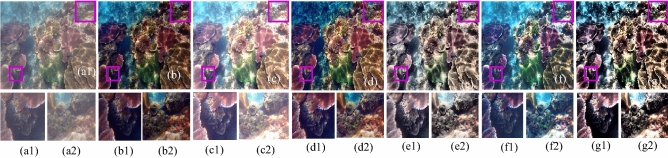
Comparison of enhancement results for underwater image “*j*”. (**a**) Input, (**b**) SDD^6^, (**c**) AGIE^25^, (**d**) PRIEN^27^, (**e**) UIE-DN^32^, (**f**) CSDNet^24^ and (**g**) Proposed method.

**Figure 12:**
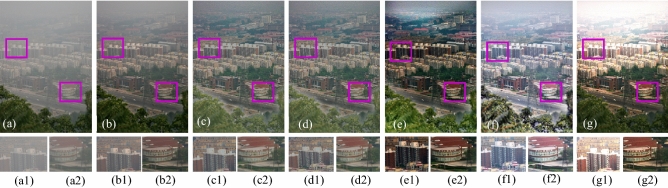
Comparison of enhancement results for hazy image “$k$”. (**a**) Input, (**b**) SDD^6^, (**c**) AGIE^25^, (**d**) PRIEN^27^, (**e**) UIE-DN^32^, (**f**) CSDNet^24^ and (**g**) Proposed method.

The visual results produced by numerous methods are shown in Figs. 4, 5, 6, 7, 8, 9, 10, 11 and 12. A narrowly scattered histograms of low illumination images are stretched by SDD to increase contrast. There are visible artefacts in flat portions when constant values of adjacent pixels are stretched out. However, our method generates artifacts images with visually satisfying appearance. Visible halo artefacts are generated by UIE-DN and CSDNet in some places as shown in halo around the bushes in image *a* (see in Fig. 5). As observed in image *b*, SRIE and PIE are unable to appropriately increase the visibility of input image (see in Fig. 9). However, the proposed approach can generally prevent halo artefacts plants in image *e* (see in Fig. 6) and the street in image *d* (see in Fig. 4). In fact, our proposed strategy and yields satisfactory results. While PRIEN aims to preserve the authenticity of images, the larger part of its output has vibrant colour. However, some elements in its results are lost such as textures of outperforms the other methods when it comes to appropriately preserving these textures. The AGIE performs worthily in illuminating dark regions. The flower in image $f1$ (see in Fig. 7) and the streetlights textures in image $g1$ are two examples of how this technique could simply over enhanced regions with relatively high intensities (see in Fig. 8). In contrast, the proposed strategy yields more realistic performance while effectively boosted perceptibility of low illumination images. In addition to the qualitative visual comparisons, we utilise quantitative metrics to evaluate an efficiency of proposed strategy. Since evaluating the quality of enhancement results in not an easy task, we utilise FSIM, entropy, VIF, EME, MAE and AB to evaluate the enhancement result comprehensively. The average FSIM, entropy, VIF, EME, MAE and AB results of the low-light input images $a, b {\text{and}} d$ are shown in Tables 1, 2 and 3 along with the results of all the comparative low-light image enhancing methods. Higher numbers indicate higher image qualities for FSIM, entropy, VIF and EME. To evaluate the distortion of the input image, FSIM compare the structural and feature similarity measurements between enhanced and input images. It is based on the gradient magnitude and phase congruency. The VIF is a full reference index for evaluating image quality that is based on both the idea of image information retrieved by human visual system and natural scene statistics. Higher EME number indicates a clearer image with more contrast and information. Information entropy or entropy is a term used to characterise the degree of randomness (or uncertainty) in a signal or an image. The average FSIM, entropy, VIF, EME, MAE and AB scores of the proposed method obtained ideal among the other techniques. Although FSIM, entropy, VIF, EME scores are marginally higher than other methods, their MAE and AB values are substantially higher than those of our technique which is ideal for better enhancing performance. The images “*g*” in Fig. 9 and “*b*” in Fig. 10 show how some of the results generated by AGIE do not appear natural when compared visually. The PRIEN cannot properly enhance the entire images (Figs. 6, 7) and produces halo artefacts. In terms of VIF and EME higher scores represent better visual contrast. Using three criteria such as signal strength mean intensity, and signal structure elements, FSIM measures perceptual differences between enhanced results and the input ones. The enhanced image quality has been deteriorated instead of improvement according to the FSIM score of lesser than 1. The proposed method which earns the maximum VIF score successfully improves the overall image quality without significantly increasing artefacts. While evaluating the quality of images, VIF measures the local features of images and global histogram thus favouring photographs with greater contrast. In addition to these images, we have presented enhancement results of medical, underwater, and dusty environment images as shown in Figs. 10, 11 and 12. In contrast, the proposed method has achieved excellent performance compared to other comparative state-of-the-art methods. We can see that the VIF scores of SDD and AGIE are low and generates poor enhancement results of the input image. We studied how well our enhancement algorithm performs in noisy cases. There is noise in this situation on other channels as well apart from the different channel. To apply the proposed method to each channel, an input image is transformed into RGB colour space. For this challenge, both of the parameters are set to 0.01. Figures 4, 5, 6, 7, 8, 9, 10, 11 and 12 displays the results of image enhancement for low-light images. The noise that is hidden under very low light conditions is extremely powerful. While PRIEN and UIE-DN can positively enhance perceptibility of low illumination photographs but they also increase intense noise. The UIE-DN is unable to illuminate the input images, and its output also exhibits obvious noise. Our approach handles low-light photographs with significant noise with satisfactory performance. We compared the findings of our technique with those from other techniques extensively. As observed in Fig. 8, when the low illumination of images by the SDD and AGIE is not restored appropriately thus numerous erroneous small structures are produced. The CSDNet generates over-enhanced result, particularly in regions having more illumination. Further, a denoising procedures inevitably blurs the entire image. Nevertheless, the performance of our technique appears more natural and sharper. Using 200 images from LIME, we examine the results of our method with that of competing techniques. Tables 1, 2 and 3 lists the average quantitative evaluation results generated from different images using several comparative methods where the best results are denoted in bold. It can be observed that, our proposed method exceeds other methods in case of all evaluation metrics. The proposed technique efficiently removes larger part of noise as demonstrated in Figs. 4, 5, 6, 7, 8, 9, 10, 11 and 12, when compares to other techniques. Similarly, other competing methods marginally blur some minute information in images while preserving some noise information in roof, wall, window, and tree. Using the objective metric FSIM, Entropy, VIF, EME, MAE and AB, we also statistically compare the results. To sum up, our method performs well for images with higher noise. Our subsequent study might involve integrating our method with noise detection, estimation techniques and automatically determining which model could be ideal for the source images.

**Table 1:** Quantitative results of a test image “$c$”.

Method	FSIM↑	VIF↑	Entropy↑	MAE↓	EME↑	AB⇓
Input image	0.8852	0.2789	5.4522	0.2342	41.9030	0.7325
SDD^6^	0.9053	0.2886	5.8027	0.2434	42.8363	0.7252
AGIE^25^	0.9172	0.3026	5.9556	0.2452	46.9828	0.6627
PRIEN^27^	0.9322	0.3108	6.3424	0.2342	48.8272	0.6262
UIE-DN^32^	0.9413	0.3244	6.7423	0.2188	53.2582	0.5625
CSDNet^24^	0.9456	0.3352	6.9082	0.2073	55.8262	0.5452
Ours	**0.9681**	**0.3560**	**7.3603**	**0.1832**	**60.7424**	**0.4880**

“$\uparrow$” represents higher the better, “$\downarrow$” represents lower the better and “$\Downarrow$” represents lowest absolute value are better.

Significant values are in bold.

**Table 2:** Quantitative results of a test image “$d$”.

	FSIM↑	VIF↑	Entropy↑	MAE↓	EME↑	AB⇓
Input image	0.8672	0.2566	5.1252	0.4027	25.7401	0.6828
SDD^6^	0.8825	0.2787	5.4524	0.3892	28.2762	0.6624
AGIE^25^	0.9062	0.2809	5.9342	0.3672	31.9227	0.6342
PRIEN^27^	0.9152	0.3065	6.1442	0.3422	34.8826	0.6277
UIE-DN^32^	0.9355	0.3197	6.5324	0.3266	38.8262	0.6026
CSDNet^24^	0.9544	0.3277	6.8026	0.3155	42.2862	0.5627
Ours	**0.9635**	**0.3491**	**7.3591**	**0.2828**	**44.7101**	**0.5357**

“$\uparrow$” represents higher the better, “$\downarrow$” represents lower the better and “$\Downarrow$” represents lower absolute value are better.

Significant values are in bold.

**Table 3:** Quantitative results for test image “*f*”.

Method	FSIM↑	VIF↑	Entropy↑	MAE↓	EME↑	AB⇓
Input image	0.8526	0.2562	5.5625	0.2829	32.0189	0.5188
SDD^6^	0.8725	0.2754	5.9725	0.2682	36.8826	0.4992
AGIE^25^	0.8926	0.2782	6.3452	0.2525	40.8622	0.4762
PRIEN^27^	0.9244	0.2892	6.6725	0.2452	45.7622	0.4425
UIE-DN^32^	0.9342	0.2982	6.9892	0.2352	56.8287	0.4153
CSDNet^24^	0.9425	0.3026	7.0234	0.2142	65.7633	0.3893
Ours	**0.9613**	**0.3201**	**7.3804**	**0.2053**	**67.7103**	**0.3680**

“$\uparrow$” represents higher the better, “$\downarrow$” represents lower the better and “$\Downarrow$” represents lowest absolute value are better.

Significant values are in bold.

### Parameter study

**Figure 13:**
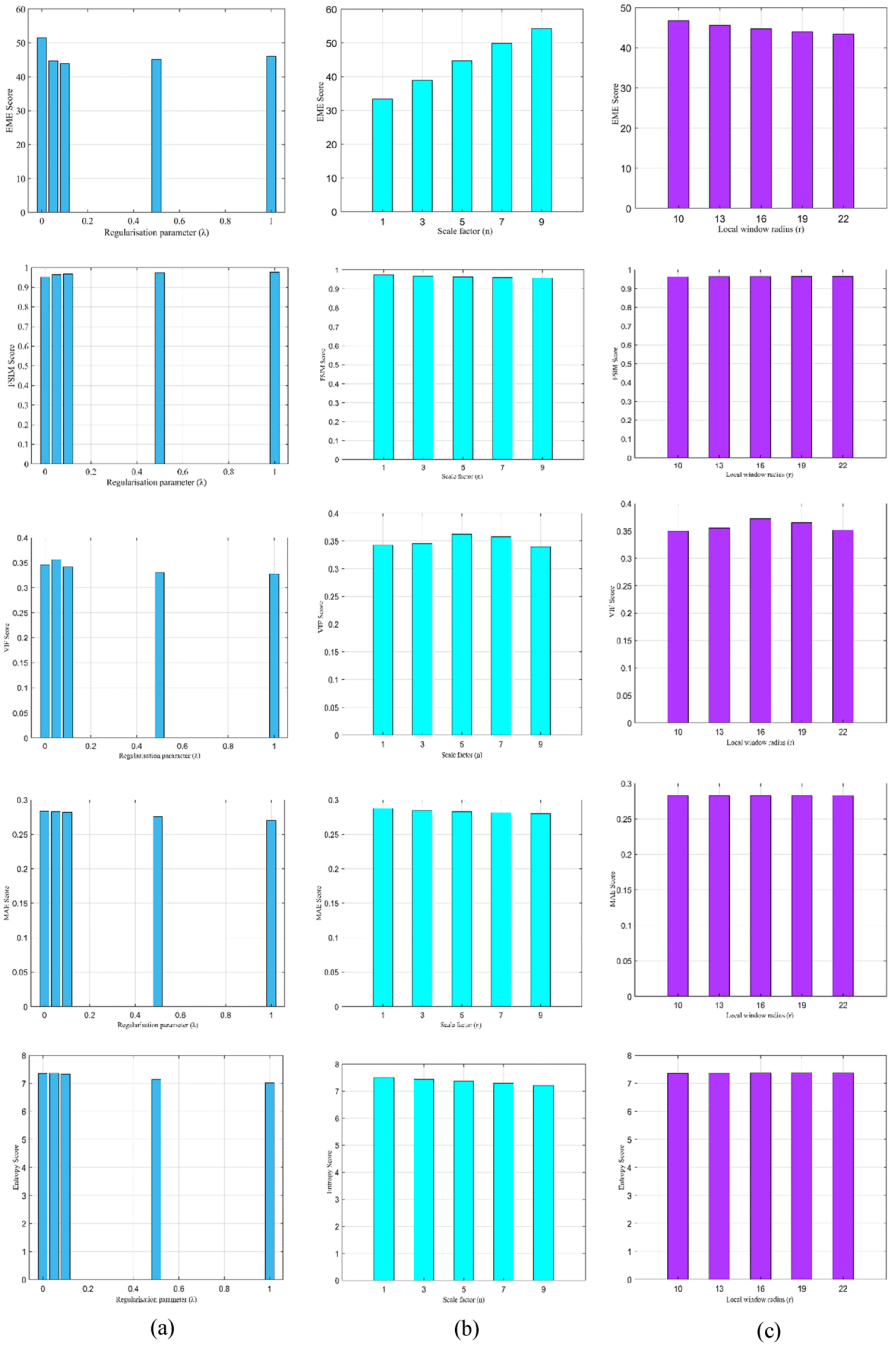
The effect of parameters; (**a**) Regularisation parameter $\lambda$, (**b**) Scale factor $n$ and (**c**) Local window radius $r$ with respect to evaluation metrics EME, FSIM, VIF, AB, MAE, and Entropy on the proposed method. Zoomed-in to have a clear viewing experience.

This section evaluates the impact of different parameters on the proposed method. In addition, we also examine how these parameters affect enhancement performance. On all of the test images, we present the quantitative results (Tables 1, 2, 3) for different images. Remember that greater FSIM, Entropy, VIF and EME values and lower MAE and AB values indicate superior visual quality. In Fig. 13, we presented multiple bar graphs representing the effects of the regularisation parameter (λ), scale factor (n) and local window radius (r) on the proposed method. We considered a regularisation parameter (λ) of (0, 0.05, 0.1, 0.5, 1). Similarly, we consider scale factor (n) of (1, 3, 5, 7, 9) and local window radius (r) of (10, 13, 16, 19, 22) in image “$c$. ” for simulations. As we can observe, performances are not consistent in terms of these parameters. In our study, we set the regularisation parameter λ, scale factor $n$ and local window radius (r) to 0.05, 5, and 16, respectively. We tried different values for these parameters during the experiments to optimise the best value. In general, the parameters limit the intensity of the noise map, sometimes smaller values oversaturate the results and higher values generate more noise. In Fig. 13, we examine the impacts of parameters in more detail, and it shows numerous valuable pieces of information. The proposed method is tested with a variety of other imaging modalities images, such as medical images, underwater or remote sensing images, as well as images captured in foggy or dusty environments to provide further validation on whether the proposed image enhancement scheme can maintain consistent performance across various lighting conditions and image content, as well as its adaptability to unknown scenarios. We can observe that, the proposed method shows consistent performance in these images with high quality enhanced results with negligible distortions and noise.

## Conclusions

This study presents a novel low-light image enhancement strategy using a camera response model and a detailed-based dictionary learning model. We sampled a large set of detail patches from high quality images to construct a training set for learning an overcomplete detail dictionary using an iterative $\ell_{1}$-norm minimization. Besides, we utilise a detail dictionary to recover an enhanced detail patches from the input image and then enhance local coherence between patches using gradient guided optimisation. To get enhanced results, we additionally recommend local exposure adjustment of low illumination images utilising cameral response models. To improve low-light images, we apply illumination estimation schemes to the selected CRM and subsequent exposure ratio maps. To examine the performance of visibility enhancement, we also proposed a reference evaluation metric and a non-reference quantitative evaluation metric. The results of the proposed technique have shown that our strategy is much better than many other existing techniques. In addition to low-light image enhancement, the proposed approach is applicable for several other similar applications, including enhancing medical images, underwater or remote sensing images, and foggy or dusty environment images. The proposed method was able to produce high quality perceptibility enhancement results, but it requires lots of processing time and memory to train the over-complete detail dictionary and reconstruct each detail patch which is its limitation. Besides, future work will focus on streamlining the training and reconstruction procedures as well as introducing some advanced techniques. Our future work will also emphasize other image and video enhancement applications.

## Data Availability

All data generated or analysed during this study are included in this published article.
